# Supplementary material for: NEK7 couples SDHB to orchestrate respiratory chain electron transport homeostasis that impedes liver fibrosis
Source: Nat Commun. 2025 Nov 28;16:10751. doi: 10.1038/s41467-025-65790-0 (PMC12663309; doi:10.1038/s41467-025-65790-0)
Supplement: Supplementary file 1 — Supplementary Information [file 41467_2025_65790_MOESM1_ESM.pdf]

SUPPLEMENTARY INFORMATION

NEK7 couples SDHB to orchestrate respiratory chain electron transport homeostasis that impedes liver fibrosis

Zhenzhen Sun[1,2,3,4,7]; Le Sun[1,3,4,7]; Hu Hua[1,3,4]; Ying Ren[1,3,4]; Wenping Zhu[1,3,4]; Xu Wang[2]; Wei Gu[2]; Songming Huang[1,3,4]; Dandan Zhong[1,5]; Ying Sun[1,5*]; Yue Zhang[1,3,4,6*]; Aihua Zhang[1,3,4*]; Zhanjun Jia[1,2,3,4,5*]

1 Nanjing Key Laboratory of Pediatrics, Children's Hospital of Nanjing Medical University, Guangzhou Road #72, Nanjing 210008, P. R. of China
2 Department of Endocrinology, Children's Hospital of Nanjing Medical University, Guangzhou Road #72, Nanjing 210008, China
3 Department of Nephrology, Children's Hospital of Nanjing Medical University, Guangzhou Road #72, Nanjing 210008, P. R. of China
4 Jiangsu Key Laboratory of Early Development and Chronic Diseases Prevention in Children, Nanjing Medical University, Nanjing 210029, P. R. of China
5 Jiangsu Key Laboratory of Geriatric Precision Medicine and Aging Intervention, Xuzhou Medical University, Xuzhou, Jiangsu 221004, P.R. China.
6 State Key Laboratory of Reproductive Medicine and Offspring Health, Nanjing Medical University, Nanjing, 211166, Jiangsu, China.
7 These authors contributed equally to this work.

*Correspondence to:
Zhanjun Jia, Nanjing Key Laboratory of Pediatrics, Children's Hospital of Nanjing Medical University, 72 Guangzhou Road, Nanjing 210008, P. R. of China, Tel: 0086-25-8311-7309, Fax: 0086-25-8330-4239, Email: jiazj72@hotmail.com.
Aihua Zhang, Department of Nephrology, Children's Hospital of Nanjing Medical University, 72 Guangzhou Road, Nanjing 210008, P. R. of China, Tel: 0086-25-8311-7309, Fax: 0086-25-8330-4239, Email: zhaihua@njmu.edu.cn.
Yue Zhang, Nanjing Key Laboratory of Pediatrics, Children's Hospital of Nanjing Medical University, 72 Guangzhou Road, Nanjing 210008, P. R. of China, Tel: 0086-25-8311-7309, Fax: 0086-25-8330-4239, Email: zyflora2006@hotmail.com.
Ying Sun, Jiangsu Key Laboratory of New Drug Research and Clinical Pharmacy, Xuzhou Medical University, Xuzhou, Jiangsu 221004, P.R. China.

content

Supplementary Figures（Fig.1-12） --- 3 - 16

Supplementary Tables (1-3) ---16 - 17

Supplementary Figures

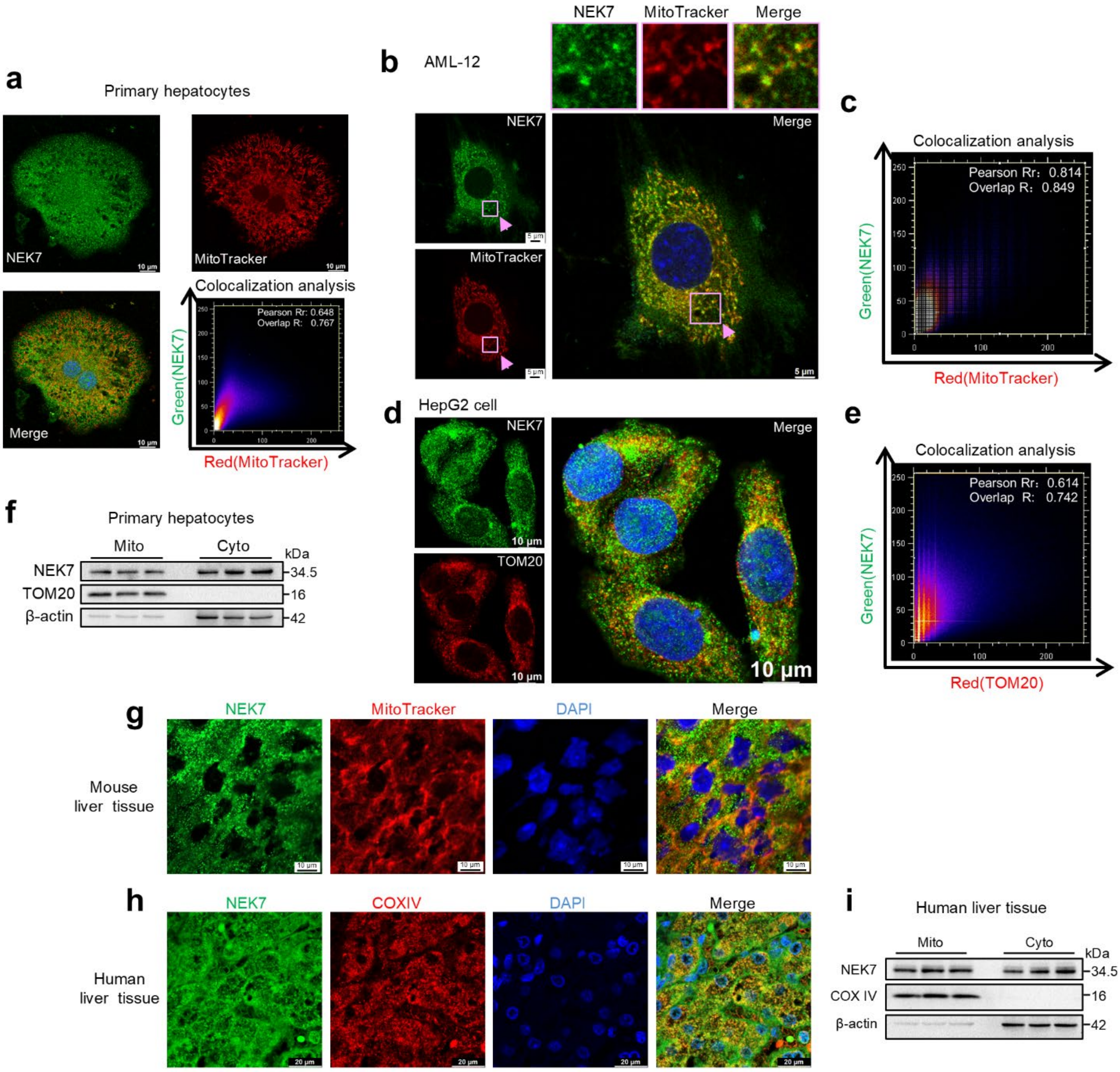

Supplementary Fig.1 Co-localization analysis of NEK7 and mitochondria

a, Representative images acquired by laser confocal microscopy depicting NEK7 distribution (Green) in the mitochondria (Red, MitoTracker) of mouse primary hepatocytes. Scale bar, 10 μm. Pearson's Rr and Overlap R analyzed by the ImageJ plugin are 0.648 and 0.767, indicating a high co-localization of NEK7 with mitochondria. N=3/group. **b**,**c,** Representative images showing localization of NEK7 (Green) in mitochondria (Red, MitoTracker) of AML-12 cells. Scale bar, 5 μm. Pearson's Rr and Overlap R (**c**) analyzed by the ImageJ plugin are 0.814 and 0.849, indicating a high co-localization of NEK7 with mitochondria. N=3/group. **d**,**e,** Representative images showing localization of NEK7 (Green) in mitochondria (Green) in the mitochondria (Red, TOM20) of HepG2 cells. Scale bar, 10 μm. Pearson's Rr and Overlap R (**e**) analyzed by the ImageJ plugin are 0.614

and 0.742, indicating a high co-localization of NEK7 with mitochondria. N=3/group. **f**, Western blotting analysis showing the NEK7 protein levels in mitochondria and cytoplasm isolated from mouse primary hepatocytes. Mito, mitochondria; Cyto, cytoplasm. **g**, Representative images of the co-localization staining of NEK7 (Green) and mitochondria (MitoTracker, Red) in the liver tissues of the wild-type mice. Scale bar, 10 μm. N=3/group. **h**, Representative images of the co-localization staining of NEK7 (Green) and mitochondria (COXIV, Red) in human liver tissues. Scale bar, 20 μm. N=5 (patient 1#-5#, shown in Supplementary information Table 1). **i**, Western blotting analysis showing the NEK7 protein levels in mitochondria and cytoplasm isolated from human liver tissues (N=3, patient 6#-8#, shown in Supplementary information Table 1). N values in **a-f** indicate biological independent replicates and N values in **g-i** indicate the numbers of mouse and human study participants. Source data are provided as a Source Data file.

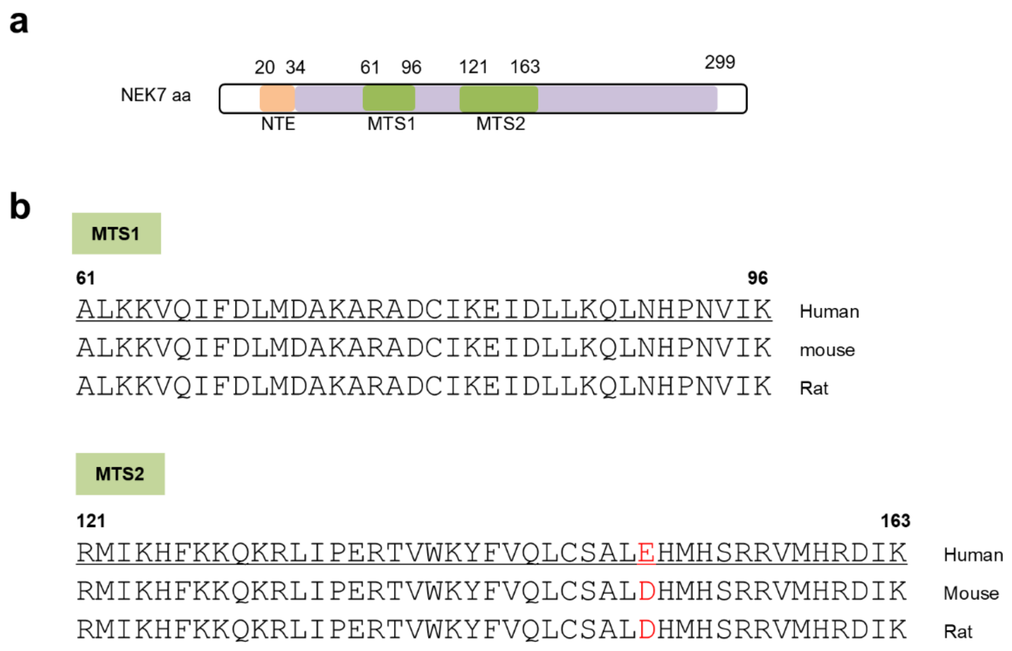

Supplementary Fig.2 Distribution and sequence conservation of the two predicted MTS peptides in NEK7 aa

a, Distribution of the two predicted MTS peptides (61-96 and 121-163) in mouse NEK7 aa. **b**, Sequences of the two MTS were highly conserved in Human, Mouse, and Rat.

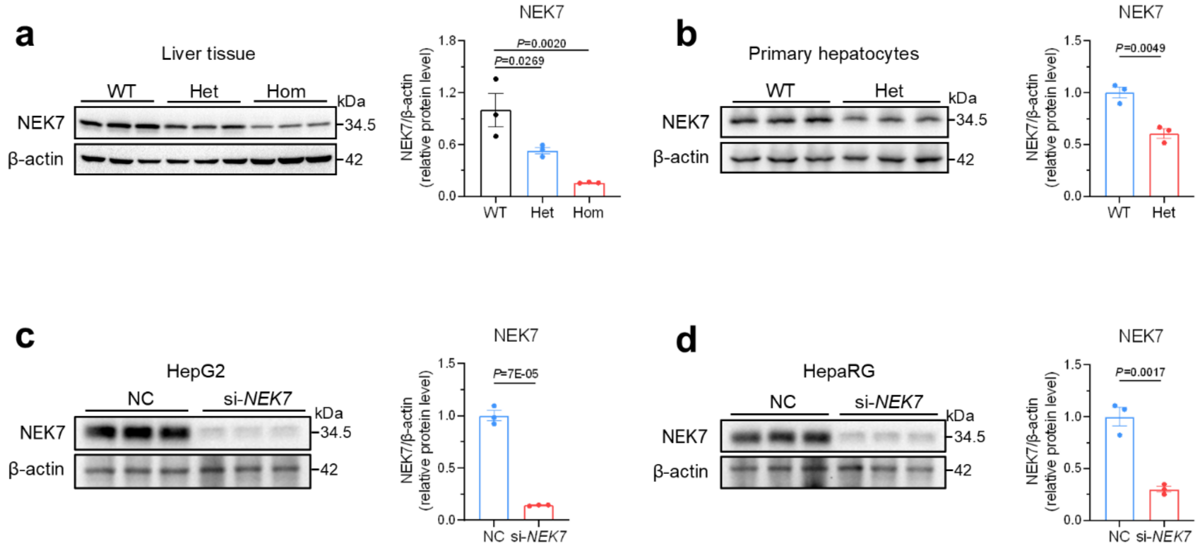

Supplementary Fig.3 Validation of NEK7 protein expression in the HepG2 cells, HepaRG cells, the liver tissues and primary hepatocytes from *NEK7* knockout mice

a,**b**, Western blotting analysis showing NEK7 expression in the liver tissues (from the WT, Het, and Hom mice) and primary hepatocytes (isolated from the livers of the WT and Het mice). N=3/group. **c**,**d**, Western blotting analysis of the level of NEK7 protein in the HepG2 (**c**) and HepaRG cells (**d**) transfected with NC or si-*NEK7* for 48 h. N=3/group. Data are presented as mean ± SE. Significant differences are analyzed using unpaired two-tailed *t*-test (**b**,**c**,**d**), and a one-way ANOVA with Benjamini multiple comparisions (**a**). N values in **a-d** indicate biological independent replicates. Source data are provided as a Source Data file.

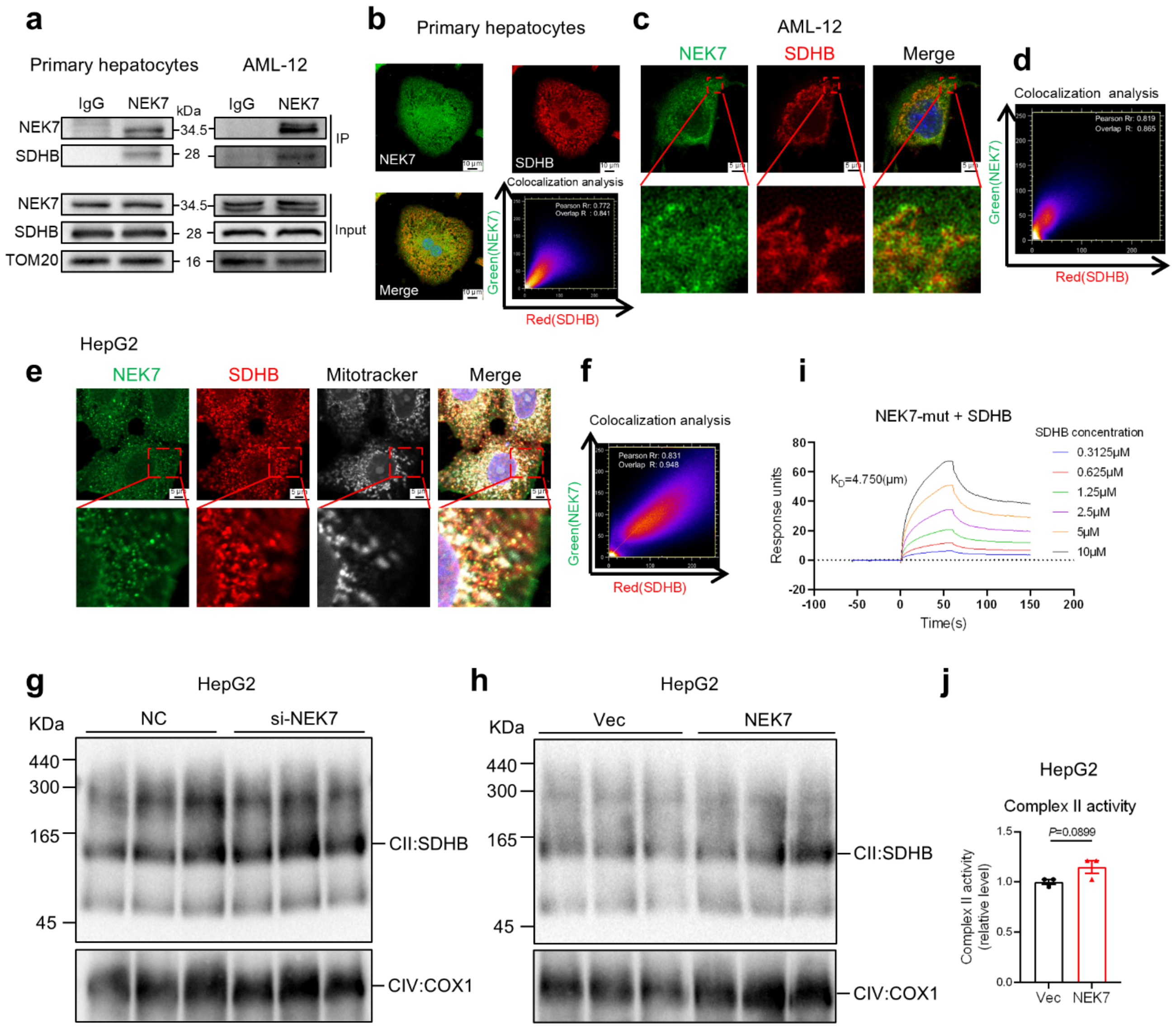

Supplementary Fig.4 Validation of the interaction of NEK7 and SDHB in hepatocytes.

a, Western blotting analysis showing the interaction of NEK7 and SDHB determined by co-immunoprecipitation with IgG and NEK7 antibody (both in AML-12 cells and mouse primary hepatocytes). **b**, Representative immunofluorescence images and the co-localization analysis show NEK7 and SDHB co-localization in mouse primary hepatocytes. Scale bar, 10 μm. N=3/group. **c**,**d**, Representative images (**c**) of immunofluorescence show NEK7 and SDHB co-localization in AML-12 cells. Scale bar, 5 μm. The ImageJ plugin conducts co-localization analysis (**d**), including Pearson's correlation Rr and Overlap R. N=3/group. **e**,**f**, Representative images (**e**) of immunofluorescence show NEK7 and SDHB co-localization in the mitochondria in HepG2 cells. Scale bar, 5 μm. The ImageJ plugin conducts co-localization analysis (**f**), including Pearson's correlation Rr and Overlap R. N=3/group.

g,h, Blue Native PAGE analysis of the mitochondrial fractions isolated from the HepG2 cells transfected with NC/si-*NEK7* or Vec/NEK7 for 48h. Complex II and complex IV was probed by anti-SDHB and anti-COX1 respectively, complex IV, as the control. N=3/group. **i**, SPR analysis showing the binding affinity of NEK7-mut and SDHB protein (Supplementary Data 2). **j,** Mitochondrial complex II activity in the HepG2 cells transfected with NEK7 or Vec for 48 h. N=3/group. Data are presented as mean ± SE. Significant differences are calculated using unpaired two-tailed *t*-test (**i**). N values in **a-h, j** indicate biological independent replicates. Source data are provided as a Source Data file.

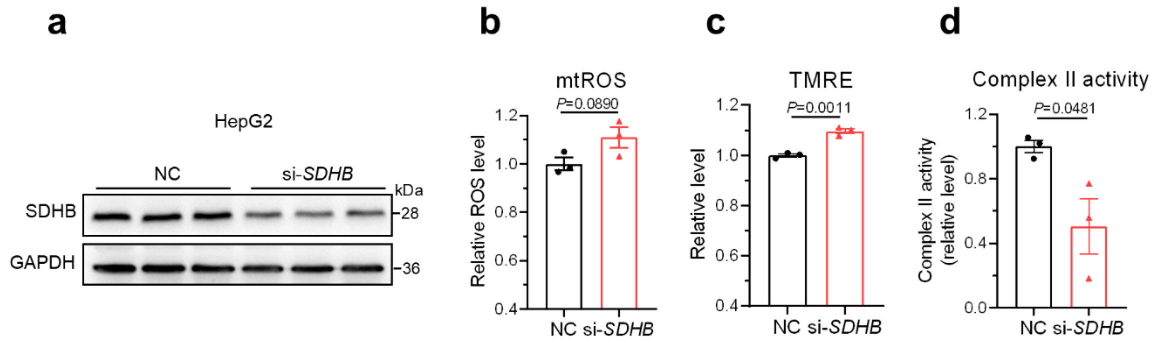

Supplementary Fig.5 Inhibiting SDHB leads to mitochondrial dysfunction

a, Western blotting analysis of the level of SDHB protein in the HepG2 cells transfected with si-*SDHB* or NC for 48 h. N=3/group. **b**, Relative mitochondrial ROS level (MitoSox Red) in the HepG2 cells (transfected with si-*SDHB*/NC for 48 h) detected by flow cytometry. N=3/group. **c**, Mitochondrial membrane potential (TMRE) in the HepG2 cells (transfected with si-*SDHB*/NC for 48 h) detected by flow cytometry. N=3/group. **d**, Mitochondrial complex II activity in the HepG2 cells transfected with si-*SDHB* or NC for 48 h. N=3/group. Data are presented as mean ± SE. Significant differences are calculated using unpaired two-tailed *t*-test (**b-d**). N values in **a-d** indicate biological independent replicates. Source data are provided as a Source Data file.

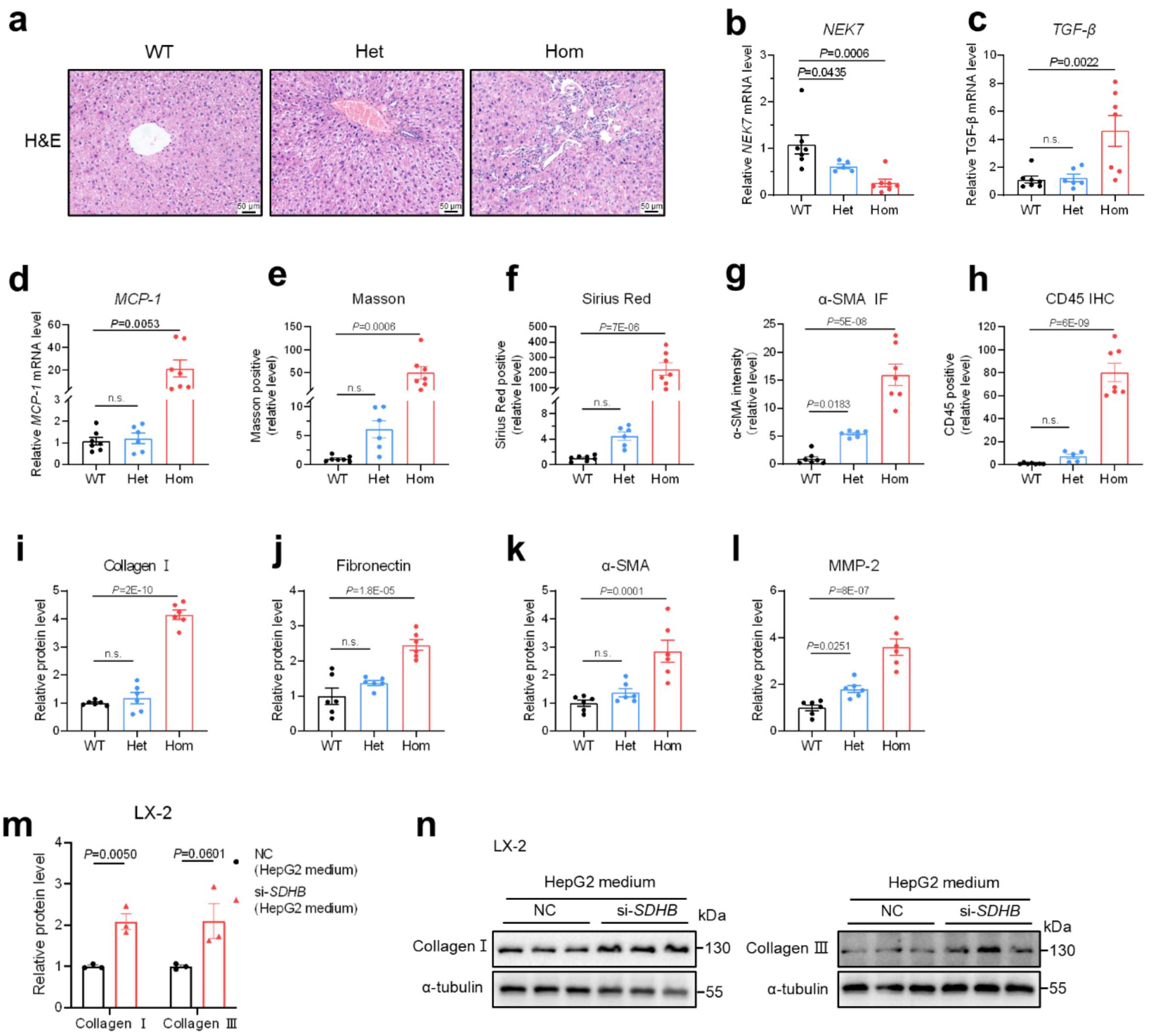

Supplementary Fig.6 Phenotype analysis of the hepatocyte *NEK7* knockout mice

a, Representative images of HE staining (scale bar, 50 μm), WT, n=7; Het, n=6; Hom, n=7. **b-d**, Relative mRNA levels of *NEK7* (WT, n=7; Het, n=5; Hom, n=7), *TGF-β* (WT, n=7; Het, n=6; Hom, n=7), and *MCP-1* (WT, n=7; Het, n=6; Hom, n=7) detected by real-time qPCR. **e-h**, Quantification of Masson, Sirius Red, α-SMA IF, and CD45 IHC staining, shown in Fig.4h. **i-l**, Quantification of the protein level of Collagen Ⅰ, Fibronectin, α-SMA, and MMP-2 in the WT, Het, and Hom mice livers showed in Fig.4r, n=6/group. **m**,**n**, The protein level of Collagen Ⅰ and Collagen Ⅲ in LX-2 cells, the LX-2 cells are treated with the conditional medium of the HepG2 cells (transfected with si-*SDHB*/NC for 48 h) for 72 h, n=3/group. Data are presented as mean ± SE. Significant differences are analyzed using unpaired two-tailed *t*-test (**m**), and a one-way ANOVA with Benjamini multiple comparisions (**b-l**), n.s., no significance. N values in **a-l** indicate the numbers of mouse and N values in **m,n** indicate biological independent replicates. Source data are provided as a Source Data file.

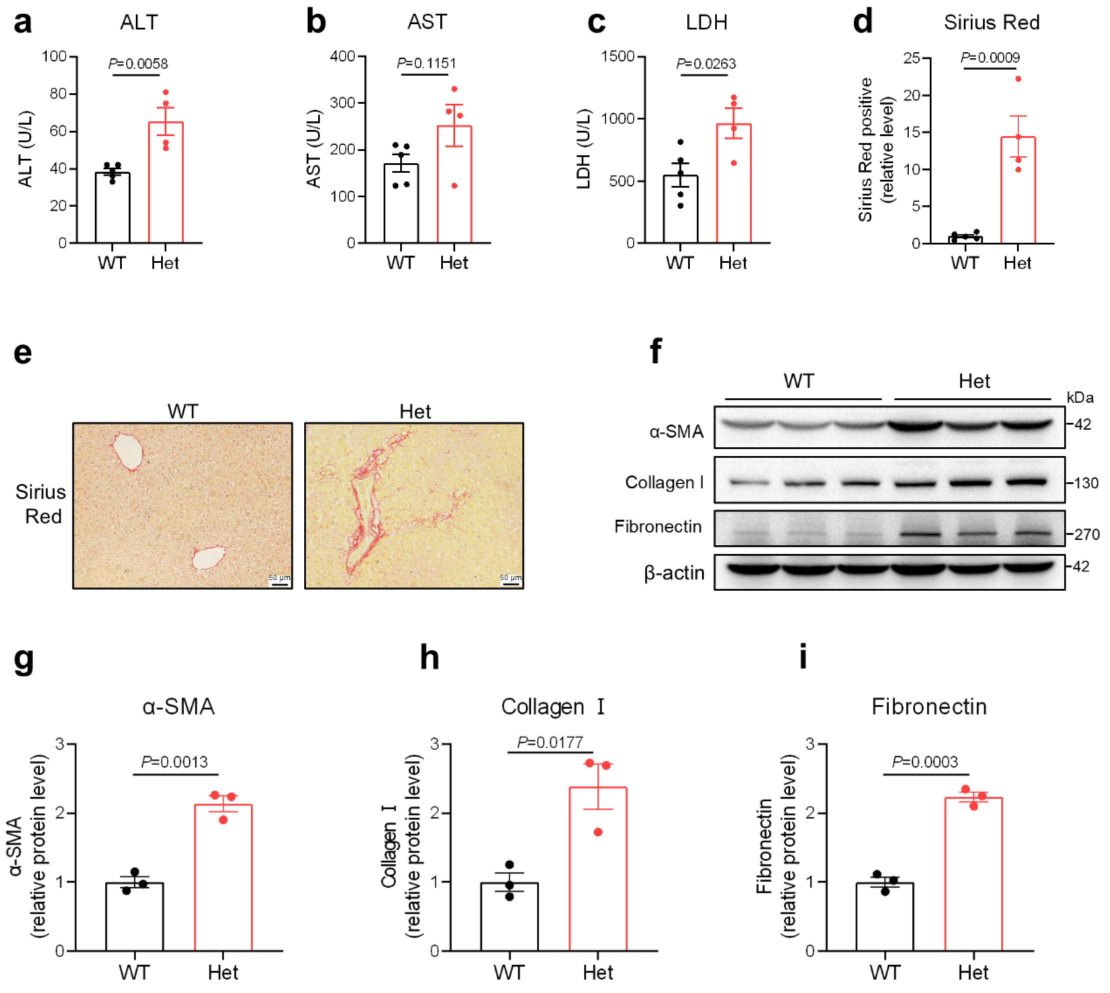

Supplementary Fig.7 Analysis of the 10-month hepatocyte *NEK7* knockout heterozygous mice

a-c, Levels of ALT, AST, and LDH in the serum of the WT (n=5) and Het (n=4) mice. **d**,**e**, Representative images and quantification of Sirius Red staining in the livers of the WT (n=5) and Het (n=4) mice. Scale bar, 50 μm. **f-i**, Western blotting analysis of α-SMA, Fibronectin, and Collagen Ⅰ expression in the WT and Het mice livers, the quantification is displayed in (**g-i**), n=3/group. Data are presented as mean ± SE. Significant differences are calculated using unpaired two-tailed *t*-test (**a-d**,**g-i**). N values in **a-i** indicate the numbers of mouse. Source data are provided as a Source Data file.

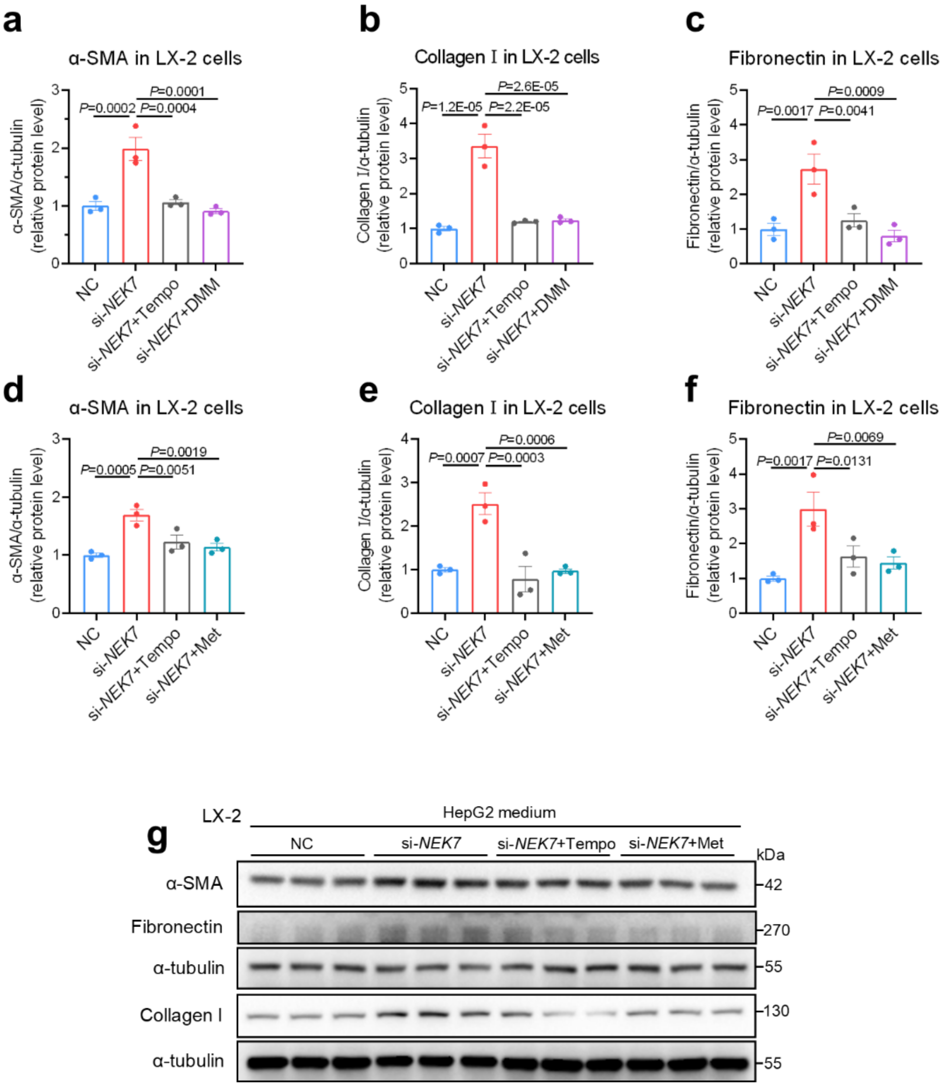

Supplementary Fig.8 Inhibiting RET and ROS alleviates the activation of LX-2 cells triggered by the HepG2 cells with NEK7 deficiency

a-**c**, Quantification of the protein level of α-SMA, Collagen Ⅰ, and Fibronectin in the LX-2 cells treated with the conditional medium of HepG2 cells. The HepG2 cells are transfected with NC or si-NEK7 for 48 h, and then treated with 500 μM Tempo or 10 μM DMM for 24 h. N= 3 biological replicates. Related to Fig.5d. **d-g**, Western blotting analysis of the expression of α-SMA, Collagen Ⅰ, and Fibronectin protein in the LX-2 cells treated with the conditional medium of HepG2 cells. The HepG2 cells are transfected with NC or si-*NEK7* for 48 h, then treated with 500 μM Tempo or 100 μM Met for 24 h. N= 3 biological replicates. Data are presented as mean ± SE. Significant differences are analyzed using a one-way ANOVA with Benjamini multiple comparisions (**a-f**). Source data are provided as a Source Data file.

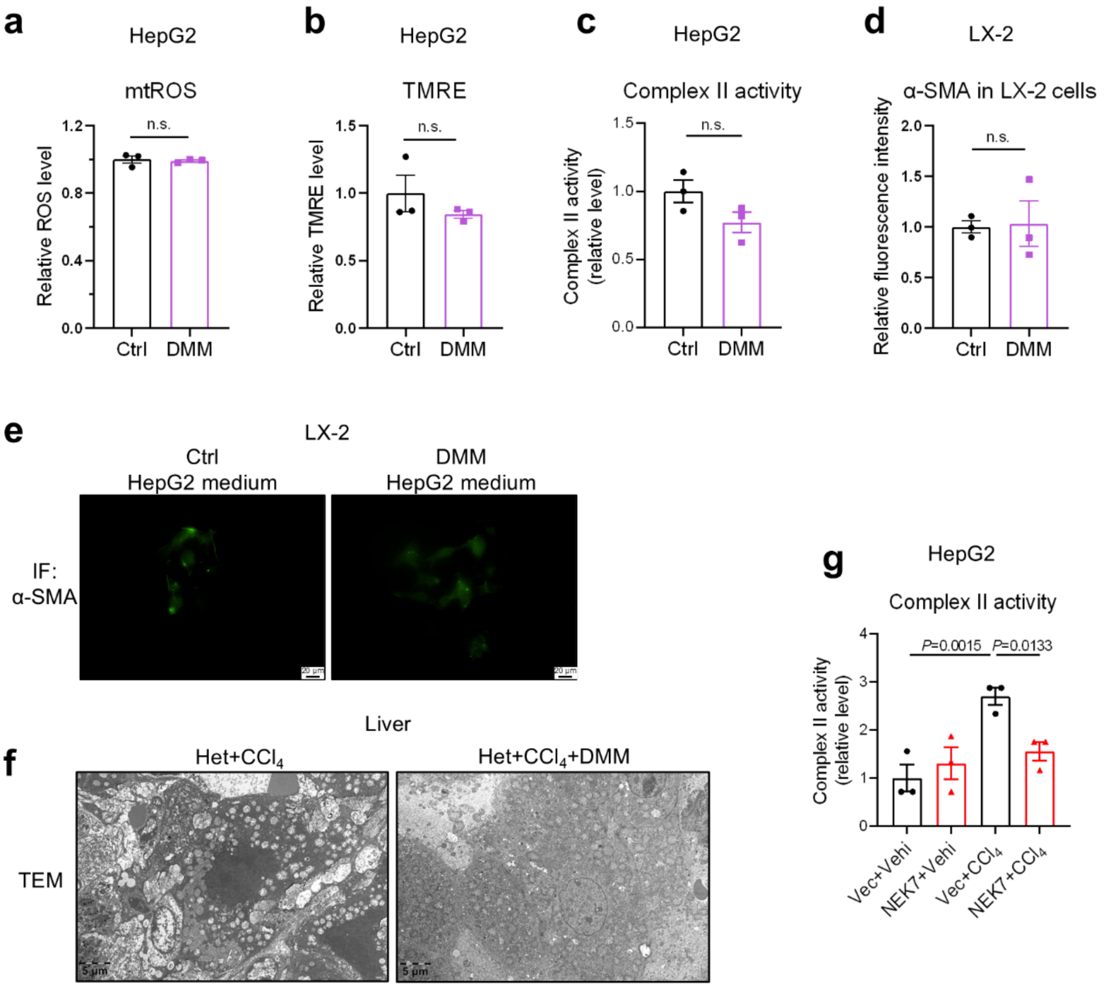

Supplementary Fig.9 Role of DMM in mitochondrial function and complex II activity in HepG2 cells.

a, Flow cytometry detected mitochondrial ROS levels (MitoSox Red) in HepG2 cells. The HepG2 cells are treated with 10 μM DMM or vehi (Ctrl) for 24 h. N=3/group. **b,** Flow cytometry detected mitochondrial membrane potential (TMRE) in control and DMM-treated HepG2 cells. N=3/group. **c**, Activity of mitochondrial complex II in the HepG2 cells treated with 10 μM DMM or vehi (Ctrl) for 24 h. N=3/group. **d**,**e**, Representative images (**e**) of α-SMA staining in LX-2 cells (scale bar, 20μm), the LX-2 cells are treated with the corresponding conditional medium of the HepG2 cells in (**a**). Relative fluorescence intensity is displayed in (**d**). N=3/group. **f,** Representative TEM images depicting the ultrastructure of the mitochondria in hepatocytes from the Het+CCl_4 and Het+CCl_4+DMM mice (scale bar, 5μm). N=3/group. **g**, Mitochondrial complex II activity in the HepG2 cells. The control (Vec) and NEK7-overexpressed HepG2 cells are treated with vehicle (Vehi) or CCl_4 (5 mM) for 24 h. N=3/group. Data are presented as mean ± SE. Significant differences are calculated using unpaired two-tailed *t*-test (**a-d**), and a one-way ANOVA with Benjamini multiple comparisions (**g**). N values in **a-e,g**

indicate biological independent replicates and N values in **f** indicate the numbers of mouse. Source data are provided as a Source Data file.

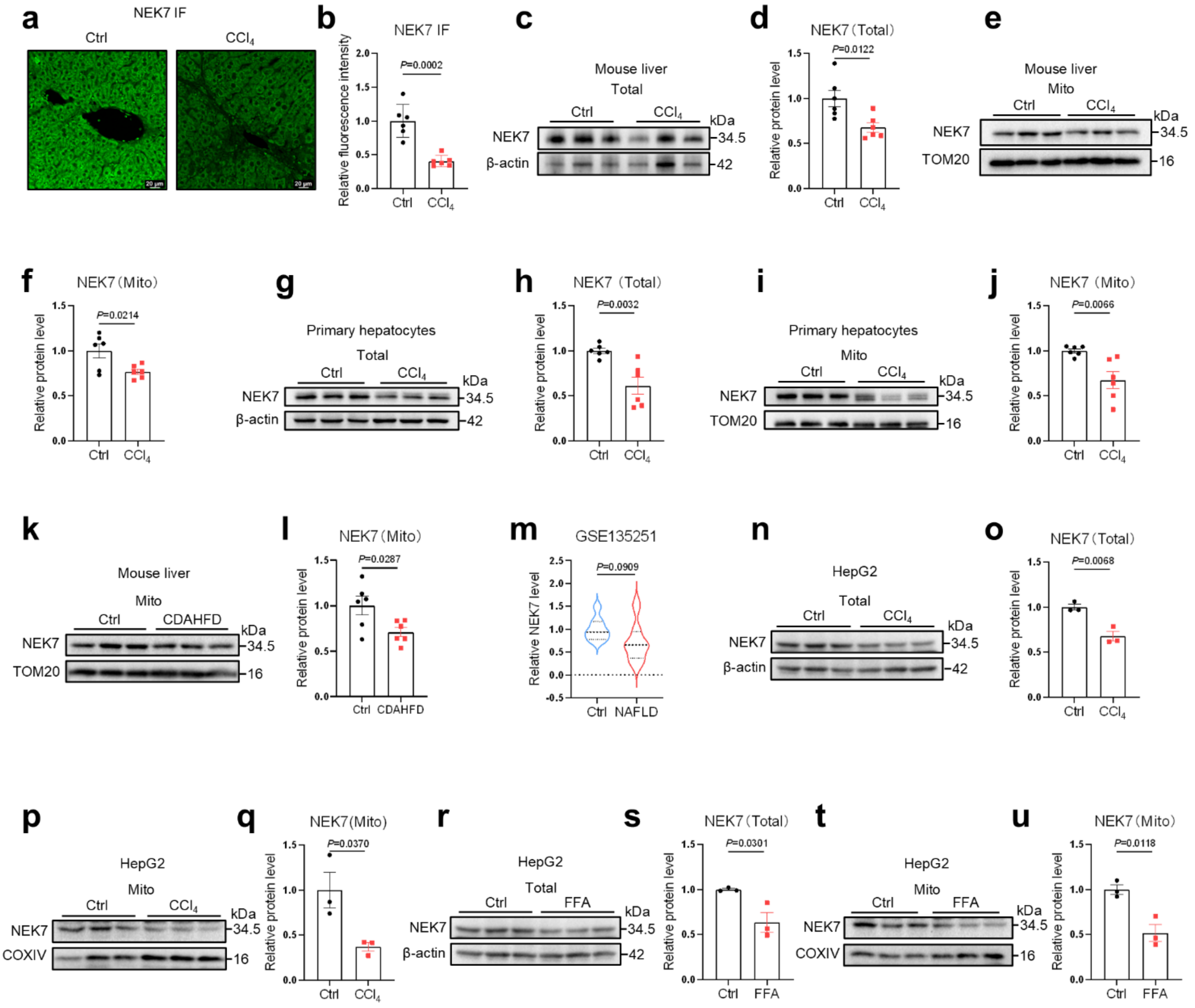

Supplementary Fig.10 Investigation of the level of NEK7 in hepatocytes and fibrotic livers.

a,b, Representative images and quantification of NEK7 IF staining (scale bar, 20 μm). N=6/group. **c-f**, Western blotting analysis showing the protein levels of NEK7 in the mouse livers of the CCl_4 model. Total, total lysates (**c,d**); Mito, mitochondrial lysates (**e,f**). The quantification is in (**d,f**). N=6/group. **g-j**, Western blotting analysis showing the protein levels of NEK7 in the mouse primary hepatocytes of the CCl_4 model. Total, total lysates (**g,h**); Mito, mitochondrial lysates (**i,j**). The

quantification is in (**h,j**). N=6/group. **k,l**, Western blotting analysis showing the protein levels of NEK7 in mitochondria from the mouse livers of the CDAHFD model. N=6/group. **m**, Relative TPM level of NEK7 in the livers from the control and NAFLD patients, from public GEO dataset GSE135251. 8 of control (fibrosis stage: 0) and 14 of NAFLD patients (fibrosis stage: 4) were included for analysis. **n-q**, Western blotting analysis showing the protein levels of NEK7 in total (**n,o**) and mitochondrial (**p,q**) lysates of the HepG2 cells treated with CCl_4 (5 mM) and the Vehicle (Ctrl) for 24h. N=3/group. **r-u**, Western blotting analysis showing the protein levels of NEK7 in total (**r,s**) and mitochondrial (**t,u**) lysates of the HepG2 cells treated with FFA (palmitic acid sodium: oleate sodium, 500 μM:500 μM) and the Vehicle (Ctrl, 1%BSA) for 24h. N=3/group. Data are presented as mean ± SE. Significant differences are analyzed using unpaired two-tailed *t*-test (**b,d,f,h,j,l,m,o,q,s,u**). N values in **a-l** indicate the numbers of mouse and N values in **n-u** indicate biological independent replicates. Source data are provided as a Source Data file.

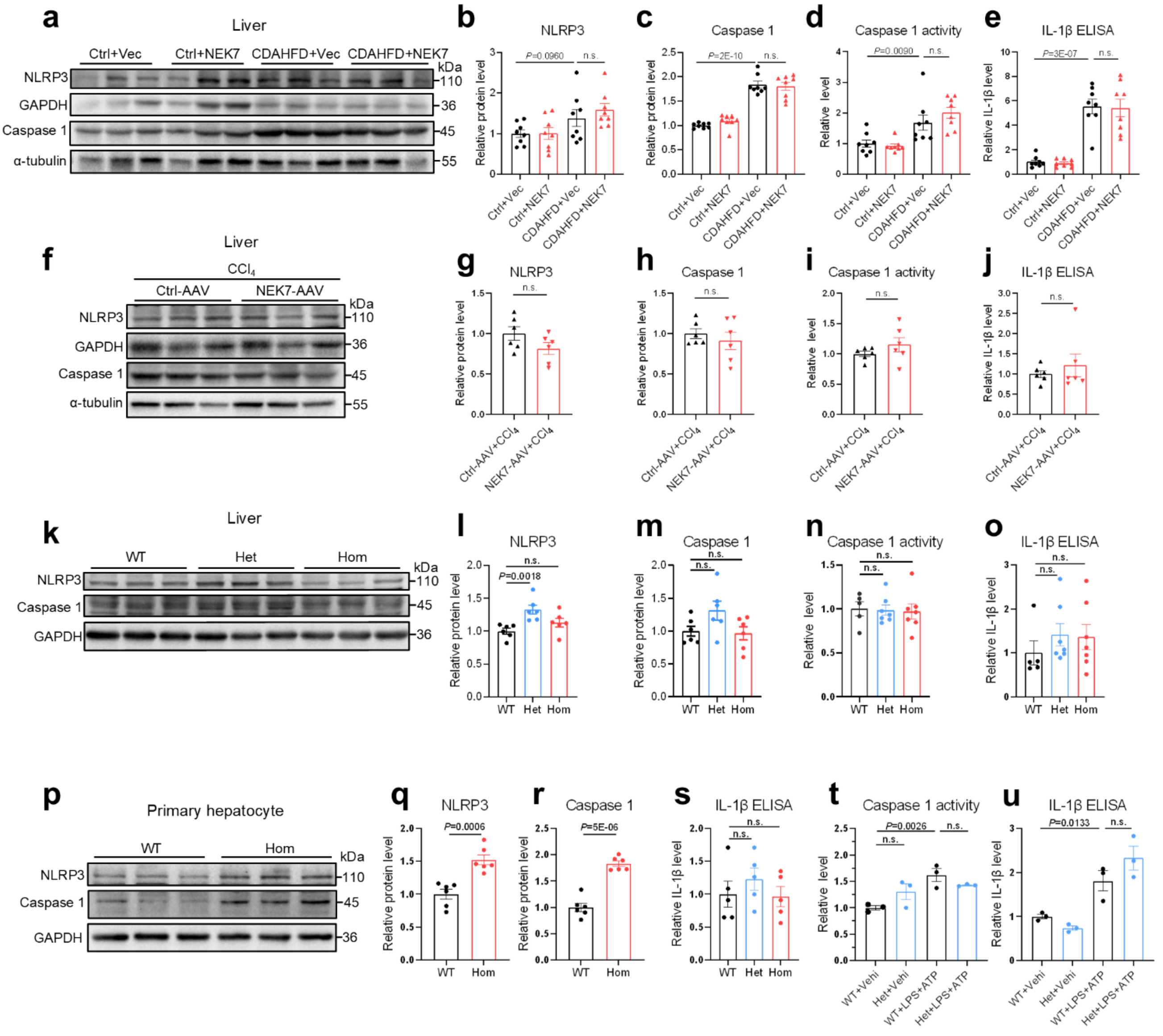

Supplementary Fig.11 Effects of NEK7 on the activation of NLRP3 inflammasome determined in diverse models.

a-c, Western blotting analysis showing the protein levels of NLRP3 and Caspase 1 in the mouse livers of the NEK7-CDAHFD model. The quantification is in (**b,c**). N=8/group. **d,** Relative level of Caspase 1 activity in the livers. N=8/group. **e,** Relative level of IL-1β in the livers tested by ELISA. N=8/group. **f-h,** Western blotting analysis showing the protein levels of NLRP3 and Caspase 1 in the mouse livers of the NEK7-AAV-CCl_4 model. The quantification is in (**g,h**). N=6/group. **i,** Relative level of Caspase 1 activity in the livers. N=6/group. **j,** Relative level of IL-1β in the livers tested by ELISA. N=6/group. **k-m,** Western blotting analysis showing the protein levels of NLRP3 and Caspase 1 in the livers from the NEK7 knockout mice. The quantification is in (**l,m**). N=6/group. **n,** Relative level of Caspase 1 activity in the livers. N=6/group. **o,** Relative level of IL-1β in the livers tested by ELISA.

N=6/group. **p-r,** Western blotting analysis showing the protein levels of NLRP3 and Caspase 1 in primary hepatocytes isolated from the WT and Hom mice. The quantification is in (**q,r**). N=6/group. **s,** Relative level of IL-1β in the primary hepatocytes (from WT, Het, Hom) tested by ELISA. N=5/group. **t,** Relative level of Caspase 1 activity in the primary hepatocytes. The primary hepatocytes isolated from the WT and Het mice livers were treated with LPS (1μg /ml) and ATP (2.5 mM) for 24 h. N=3/group. **u,** Relative level of IL-1β in the medium of the primary hepatocytes tested by ELISA. The hepatocytes were treated as shown in (**t**). N=3/group. Data are presented as mean ± SE. Significant differences are analyzed using unpaired two-tailed *t*-test (**g-j,q,r**), and a one-way ANOVA with Benjamini multiple comparisions (**b-e,l-o,s-u**). N values in **a-o** indicate the numbers of mouse and N values in **p-u** indicate biological independent replicates. Source data are provided as a Source Data file.

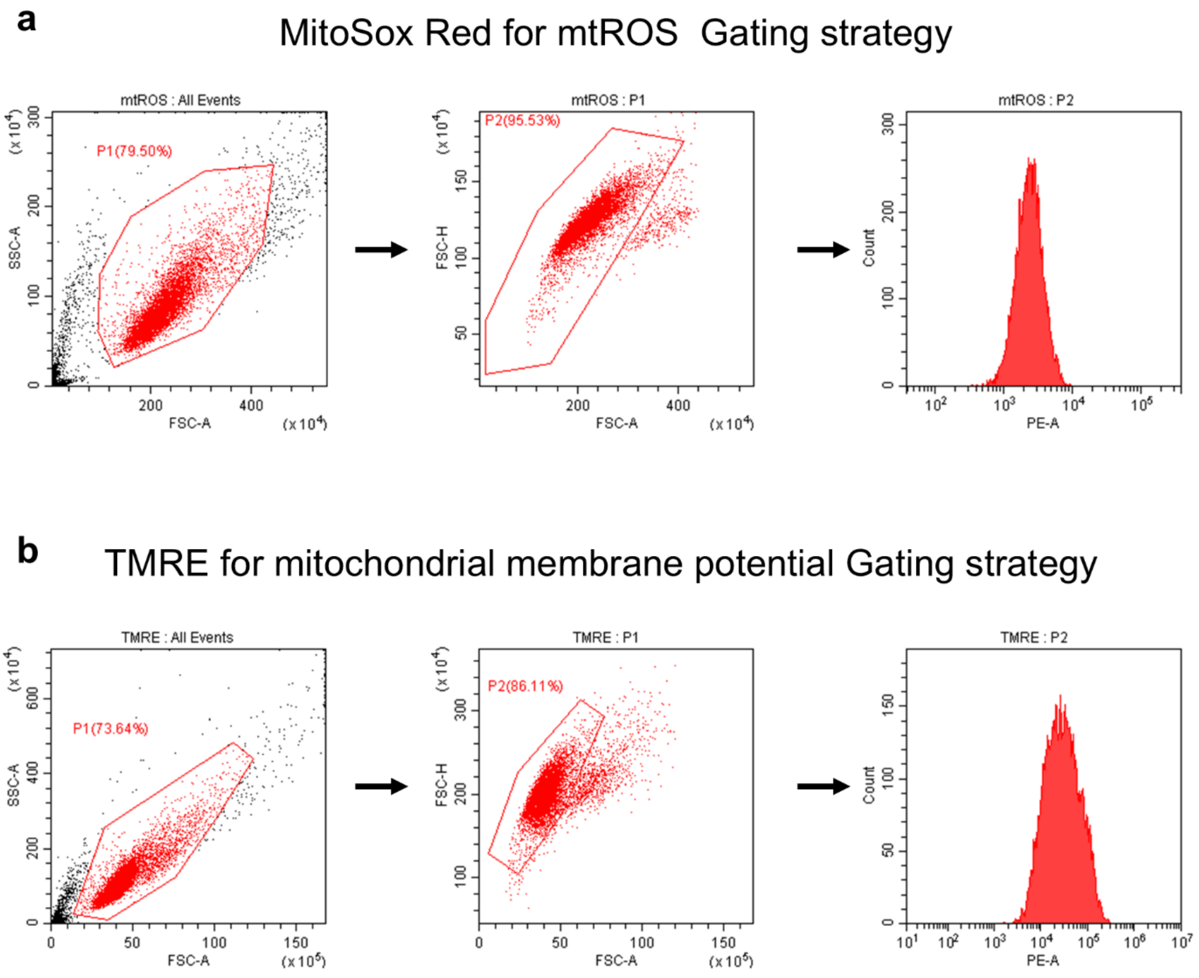

Supplementary Fig.12. Flow cytometry gating strategies.

a, Mitochondrial ROS were detected using MitoSox Red by Flow cytometry. **b,** Mitochondrial membrane potential was measured using TMRE by Flow cytometry. The FSC-A/SSC-A gating was used to determine the main live cell population. FSC-A/FSC-H gating was used

to exclude the doublets. Finally, the corresponding fluorescent signals (PE or FITC-Count) were collected for statistical analysis. The strategy is representative for three independent experiments.

Supplementary Tables

Supplementary Table 1. Clinical information of the human samples used in this study

Patients	Sex	Age	Region in the liver	Disease
1#	Female	5 years and 9 months	Normal tissue adjacent to the tumor	Hepatoblastoma
2#	Female	5 years and 4 months	Normal tissue adjacent to the tumor	Hepatoblastoma
3#	Female	1 year and 5 months	Normal tissue adjacent to the tumor	Hepatoblastoma
4#	Female	2 years	Normal tissue adjacent to the tumor	Hepatoblastoma
5#	Male	3 years and 5 months	Normal tissue adjacent to the tumor	Hepatoblastoma
6#	Female	6 years and 5 months	Normal tissue adjacent to the tumor	Hepatoblastoma
7#	Female	9 years and 10 months	Normal tissue adjacent to the tumor	Hepatoblastoma
8#	Male	7 years and 6 months	Normal tissue adjacent to the tumor	Hepatoblastoma

Supplementary Table 2. Real-time qPCR primer information

Gene Name	Sequence<br>(F, Forward primer; R, Reverse primer)	Supplier
Human *NEK7*	F: GCCTTACGACCGGATATGGG<br>R: CACTAAATTGTCCGCGACCAA	Tsingke Biotechnology
Human *GAPDH*	F: ACAACTTTGGTATCGTGGAAGG<br>R: GCCATCACGCCACAGTTTC	Tsingke Biotechnology
Human *Collagen III*	F: TTGAAGGAGGATGTTCCCATCT<br>R: ACAGACACATATTTGGCATGGTT	Tsingke Biotechnology
Human *TIMP-1*	F: CTTCTGCAATTCCGACCTCGT<br>R: ACGCTGGTATAAGGTGGTCTG	Tsingke Biotechnology
Mouse *Collagen I*	F: TAAGGGTCCCCAATGGTGAGA<br>R: GGGTCCCTCGACTCCTACAT	Tsingke Biotechnology
Mouse *Collagen III*	F: CAGGACCTAAGGGCGAAGATG<br>R: TCCGGGCATACCCCGTATC	Tsingke Biotechnology
Mouse *Fibronectin*	F: ATGTGGACCCCTCCTGATAGT<br>R: GCCCAGTGATTTCAGCAAAGG	Tsingke Biotechnology
Mouse *TIMP-1*	F: CTTGGTTCCCTGGCGTACTC<br>R: ACCTGATCCGTCCACAAACAG	Tsingke Biotechnology
Mouse	F: CCCAGACATCAGGGAGTAATGG	Tsingke

α-SMA	R: TCTATCGGATACTTCAGCGTCA	Biotechnology
Mouse<br>*MMP-2*	F: CAAGTTCCCCGGCGATGTC<br>R: TTCTGGTCAAGGTCACCTGTC	Tsingke<br>Biotechnology
Mouse<br>*IL-6*	F: TAGTCCTTCCTACCCCAATTTCC<br>R: TTGGTCCTTAGCCACTCCTTC	Tsingke<br>Biotechnology
Mouse<br>*IL-1β*	F: GAAATGCCACCTTTTGACAGTG<br>R: TGGATGCTCTCATCAGGACAG	Tsingke<br>Biotechnology
Mouse<br>*TNF-α*	F: CAGGCGGTGCCTATGTCTC<br>R: CGATCACCCCGAAGTTCAGTAG	Tsingke<br>Biotechnology
Mouse<br>*TGF-β*	F: TACGCCTGAGTGGCTGTCTT<br>R: CGTGGAGTTTGTTATCTTTGCT	Tsingke<br>Biotechnology
Mouse<br>*MCP-1*	F: TTAAAAACCTGGATCGGAACCAA<br>R: GCATTAGCTTCAGATTTACGGGT	Tsingke<br>Biotechnology
Mouse<br>*GAPDH*	F: CATCACTGCCACCCAGAAGACTG<br>R: ATGCCAGTGAGCTTCCCGTTCAG	Tsingke<br>Biotechnology
18S RNA	F: ACGGACAGGATTGACAGA<br>R: CGCTCCACCAACTAAGAA	Tsingke<br>Biotechnology

Supplementary Table 3. Target sequences of the siRNAs used in this paper.

**siRNAs**	**Target sequences**	**Supplier**
si-*NEK7*<br>(human)	1#：GAGTACCAGTAGCTTTAAA<br>2#：CACCTATGTTTATGACGTA<br>3#：CTCCGACAGTTAGTTAATA	RIBOBIO<br>technology
si-*SDHB*<br>(human)	1#：GGCCTGCAGTTCTTATGCA<br>2#：GGATGATTGACTCCAGAGA<br>3#：CCCGAAGGATTGACACCAA	RIBOBIO<br>technology
