# Supplementary material for: NEK7 couples SDHB to orchestrate respiratory chain electron transport homeostasis that impedes liver fibrosis
Source: Nat Commun. 2025 Nov 28;16:10751. doi: 10.1038/s41467-025-65790-0 (PMC12663309; doi:10.1038/s41467-025-65790-0)
Supplement: Supplementary file 2 — Description of Additional Supplementary Files [file 41467_2025_65790_MOESM2_ESM.pdf]

Description of Supplementary Data Files

File Name: Supplementary Data 1
Description: The full list of NEK7 interacting proteins (including NEK7-proteins, NEK7_unique_proteins compared with the IgG result, and the total 68 of NEK7-IP related proteins in energy production and conversion of the metabolism module) from the mass spectrometry analysis.

File Name: Supplementary Data 2
Description: The data of Surface Plasmon Resonance (SPR) analysis (including proteins NEK7+SDHB SPR in Fig.3l and proteins NEK7-mut+SDHB SPR in Supplementary Fig.4i).

File Name: Supplementary Data 3
Description: The data of Rg (Radius of gyration) value of SDH complex after SDHB binding with NEK7 generated by molecular dynamics simulation (Fig.3m of the main figures).

File Name: Supplementary Data 4
Description: Figure 1 shows the RMSDs curves of NEK7 and each subunit in the SDH complex as evidence for evaluating the final equilibrium state of the simulation. Table 1 is an information summary list of molecular dynamics simulation.

File Name: Supplementary Data 5
Description: The data of the RMSDs values of NEK7 and each subunit in the SDH complex in Figure 1 in Supplementary Data 4.

File Name: Supplementary Data 6
Description: The final trajectory file of the molecular dynamics simulation.
