# Supplementary material for: NEK7 couples SDHB to orchestrate respiratory chain electron transport homeostasis that impedes liver fibrosis
Source: Nat Commun. 2025 Nov 28;16:10751. doi: 10.1038/s41467-025-65790-0 (PMC12663309; doi:10.1038/s41467-025-65790-0)
Supplement: Supplementary file 4 — Reporting Summary [file 41467_2025_65790_MOESM4_ESM.pdf]

nature portfolio

Corresponding author(s): Zhanjun Jia

Last updated by author(s): Sep 30, 2025

Reporting Summary

Nature Portfolio wishes to improve the reproducibility of the work that we publish. This form provides structure for consistency and transparency in reporting. For further information on Nature Portfolio policies, see our Editorial Policies and the Editorial Policy Checklist.

Statistics

For all statistical analyses, confirm that the following items are present in the figure legend, table legend, main text, or Methods section.

n/a	Confirmed	
☐	☒	The exact sample size (*n*) for each experimental group/condition, given as a discrete number and unit of measurement
☐	☒	A statement on whether measurements were taken from distinct samples or whether the same sample was measured repeatedly
☐	☒	The statistical test(s) used AND whether they are one- or two-sided<br>*Only common tests should be described solely by name; describe more complex techniques in the Methods section.*
☒	☐	A description of all covariates tested
☐	☒	A description of any assumptions or corrections, such as tests of normality and adjustment for multiple comparisons
☐	☒	A full description of the statistical parameters including central tendency (e.g. means) or other basic estimates (e.g. regression coefficient) AND variation (e.g. standard deviation) or associated estimates of uncertainty (e.g. confidence intervals)
☐	☒	For null hypothesis testing, the test statistic (e.g. *F*, *t*, *r*) with confidence intervals, effect sizes, degrees of freedom and *P* value noted<br>*Give P values as exact values whenever suitable.*
☒	☐	For Bayesian analysis, information on the choice of priors and Markov chain Monte Carlo settings
☒	☐	For hierarchical and complex designs, identification of the appropriate level for tests and full reporting of outcomes
☐	☒	Estimates of effect sizes (e.g. Cohen's *d*, Pearson's *r*), indicating how they were calculated

Our web collection on statistics for biologists contains articles on many of the points above.

Software and code

Policy information about availability of computer code

Data collection	ImageJ (1.54g), LAS_X_Core (3.9.0_28093), ZEN (2012), Wave pro (10.1.0.1), CytExpert (2.3.1.22), LightCycler 96 (1.1.0.1320), QuantStudio 3 (V1.4), Image Lab (6.0.1), K-Viewer (1.5.3.1).
Data analysis	ImageJ (1.54g), CytExpert (2.3.1.22), Wave pro (10.1.0.1), GraphPad Prism (10.0).

For manuscripts utilizing custom algorithms or software that are central to the research but not yet described in published literature, software must be made available to editors and reviewers. We strongly encourage code deposition in a community repository (e.g. GitHub). See the Nature Portfolio guidelines for submitting code & software for further information.

Data

Policy information about availability of data

All manuscripts must include a data availability statement. This statement should provide the following information, where applicable:

- Accession codes, unique identifiers, or web links for publicly available datasets
- A description of any restrictions on data availability
- For clinical datasets or third party data, please ensure that the statement adheres to our policy

The raw RNA sequencing data generated in this study have been deposited at the NCBI GEO dataset (accession numbers: GSE272141) and can be accessed at https://www.ncbi.nlm.nih.gov/geo/query/acc.cgi?acc=GSE272141. The mass spectrometry data generated in this study have been deposited at ProteomeXchange Repository (project number: PXD068758) and can be accessed at https://proteomecentral.proteomexchange.org/cgi/GetDataset?ID=PXD068758. The full list of NEK7 interacting proteins (including the total 68 of NEK7-IP related proteins in energy production and conversion of the metabolism module) from the mass

spectrometry analysis were provided in Supplementary Data 1. All other data are included in the Supplementary information files and Source data file. Source data are provided with this paper.

Research involving human participants, their data, or biological material

Policy information about studies with human participants or human data. See also policy information about sex, gender (identity/presentation), and sexual orientation and race, ethnicity and racism.

Reporting on sex and gender	The human liver samples from both sex of hepatoblastoma patients were used. The information on sex of each individual was provided in Source Data and Supplementary Information.
Reporting on race, ethnicity, or other socially relevant groupings	n/a
Population characteristics	Clinical information of the patients are listed in Supplementary Table 1 of Supplementary Information.
Recruitment	In this study, 1) liver sections and tissues from the normal liver tissues adjacent to the hepatoblastoma of patients were included at Children's Hospital of Nanjing Medical University, China. Total of 8 samples were collected from the patients who were diagnosed with hepatoblastoma at Children's Hospital of Nanjing Medical University. 2) We analyzed the RNA-seq data in public GEO dataset (GSE135251) to identify the level of NEK7 in fibrotic livers of NAFLD patients. The NAFLD patients with higher level of liver fibrosis (fibrosis stage:4, n=14) and the control with no liver fibrosis (fibrosis stage:0, n=8) were included for analysis.
Ethics oversight	This study was conducted in accordance with the ethical principles described in the Declaration of Helsinki. The study protocol with the liver samples in this study was approved by the Committee on Clinical Research Ethics of Children's Hospital of Nanjing Medical University and informed consent was obtained from all participants (or their parents/guardians).

Note that full information on the approval of the study protocol must also be provided in the manuscript.

Field-specific reporting

Please select the one below that is the best fit for your research. If you are not sure, read the appropriate sections before making your selection.

☒ Life sciences ☐ Behavioural & social sciences ☐ Ecological, evolutionary & environmental sciences

For a reference copy of the document with all sections, see nature.com/documents/nr-reporting-summary-flat.pdf

Life sciences study design

All studies must disclose on these points even when the disclosure is negative.

Sample size	Sample sizes were provided within each figure legend.<br>Sample sizes determination were based on the experiences from our lab and others with similar experiments in the past.<br>No statistical methods were used to predetermine sample size.
Data exclusions	No data were excluded from analyses.
Replication	For the in vitro experiments, the sample size is generally more than 3 independent biological replicates per group for statistical analysis, while in vivo experiments, the number of mice is generally more than 5 per group for statistical analysis.
Randomization	Sex- and aged- matched control and experimental mice were included according to their genotypes. All mice were randomly allocated in groups.
Blinding	Group allocation, data collection and analysis were unbiased.

Reporting for specific materials, systems and methods

We require information from authors about some types of materials, experimental systems and methods used in many studies. Here, indicate whether each material, system or method listed is relevant to your study. If you are not sure if a list item applies to your research, read the appropriate section before selecting a response.

Materials & experimental systems

n/a	Involved in the study
☐	☒ Antibodies
☐	☒ Eukaryotic cell lines
☒	☐ Palaeontology and archaeology
☐	☒ Animals and other organisms
☒	☐ Clinical data
☒	☐ Dual use research of concern
☒	☐ Plants

Methods

n/a	Involved in the study
☒	☐ ChIP-seq
☐	☒ Flow cytometry
☒	☐ MRI-based neuroimaging

Antibodies

Antibodies used

Antibodies used for western blot and BN page:
Anti-NEK7,Abcam,ab133514, EPR4900 (1:1000);
Anti-COXIV,Proteintech,Cat No.66110-1-Ig, 2A7B2 (1:1000);
Anti-GAPDH,Proteintech,Cat No. 10494-1-AP (1:1000);
Anti-SDHB,Proteintech,Cat No.67600-1-Ig, 1A2A5 (1:1000);
Anti-α-SMA,HUABIO,ET1607-53 (1:1000);
Anti-Fibronectin,Proteintech,Cat No. 15613-1-AP (1:1000);
Anti-MMP-2,Proteintech,Cat No. 10373-2-AP (1:1000);
Anti-Collagen I,BEIJING BIOSYNTHESIS,bs-10423R (1:1000);
Anti-Collagen III,BEIJING BIOSYNTHESIS ,bs-0549R (1:1000);
Anti-β-actin,Proteintech,Cat No.66009-1-Ig, 2D4H5 (1:1000);
Anti-α-tubulin,Proteintech,Cat No.66031-1-Ig, 1E4C11 (1:1000);
Goat anti-rabbit IgG-HRP,Beyotime,Cat A0208 (1:1000);
Goat anti-mouse IgG-HRP,Beyotime,Cat A0216 (1:1000);
Pan Phospho-Serine/Threonine, Abmart, T91067S (1:1000);
ubiquitin Polyclonal antibody, Proteintech, Cat No. 10201-2-AP (1:1000);
Anti-NLRP3, Proteintech, Cat No. 19771-1-AP (1:1000);
Anti-Caspase 1, Proteintech, Cat No. 22915-1-AP (1:1000);
Anti-COX1, HUABIO, Catalog# HA722838 (1:1000);

Antibodies used for CO-IP:
Anti-IgG,Proteintech,Cat No.30000-0-AP (4μg/sample);
Anti-NEK7,Abcam,ab133514 (4μg/sample);

Antibodies used for IHC/IF:
Anti-NEK7,BEIJING BIOSYNTHESIS,bs-7758R (1:500);
Anti-Flag,Sigma,F1804 (1:500);
Anti-EGFP,BEIJING BIOSYNTHESIS,bs-2194R (1:500);
Anti-TOM20,ABclonal,A16896 (1:500);
Anti-CD45,BEIJING BIOSYNTHESIS,bs-4819R (1:500);
Anti-4-HNE (4-Hydroxynonenal),R&D,Cat No.MAB3249 (1:500);
Alexa Fluor 488-labeled goat anti-rabbit IgG,Beyotime,A0423 (1:200);
Alexa Fluor 647-labeled goat anti-mouse IgG,,Beyotime,A0473 (1:200);

Validation

All antibodies are commercially available. They are all well-validated by previous studies referred to the manufacturer's website.
Anti-NEK7,Abcam,ab133514 https://www.abcam.cn/products/primary-antibodies/nek7-antibody-epr4900-ab133514
Anti-COXIV,Proteintech,Cat No.66110-1-Ig;
https://www.ptgcn.com/products/COXIV-Antibody-66110-1-Ig.htm
Anti-GAPDH,Proteintech,Cat No. 10494-1-AP;
https://www.ptgcn.com/products/GAPDH-Antibody-10494-1-AP.htm
Anti-SDHB,Proteintech,Cat No.67600-1-Ig;
https://www.ptgcn.com/products/SDHB-Antibody-67600-1-Ig.htm
Anti-α-SMA,HUABIO,ET1607-53;
https://huabio.cn/products/alpha-smooth-muscle-Actin-antibody-ET1607-53
Anti-Fibronectin,Proteintech,Cat No. 15613-1-AP;
https://www.ptgcn.com/products/FN1-Antibody-15613-1-AP.htm
Anti-MMP-2,Proteintech,Cat No. 10373-2-AP;
https://www.ptgcn.com/products/MMP2-Antibody-10373-2-AP.htm
Anti-Collagen I,BEIJING BIOSYNTHESIS,bs-10423R;
https://www.biosschina.com/#/productDetail?goods_id=8236
Anti-Collagen III,BEIJING BIOSYNTHESIS ,bs-0549R;
https://www.biosschina.com/#/productDetail?goods_id=22422

Anti-β-actin,Proteintech,Cat No. 66009-1-Ig;
https://www.ptgcn.com/products/Pan-Actin-Antibody-66009-1-Ig.htm
Anti-α-tubulin,Proteintech,Cat No.66031-1-Ig;
https://www.ptgcn.com/products/tubulin-Alpha-Antibody-66031-1-Ig.htm
Goat anti-rabbit IgG-HRP,Beyotime,Cat A0208;
https://m.beyotime.com/mobilegoods.do?method=code&code=A0208
Goat anti-mouse IgG-HRP,Beyotime,Cat A0216;
https://m.beyotime.com/mobilegoods.do?method=code&code=A0216
Pan Phospho-Serine/Threonine, Abmart, T91067S;
https://www.ab-mart.com.cn/product.aspx?id=3&f=cn&keys=T91067S
ubiquitin Polyclonal antibody, Proteintech, Cat No. 10201-2-AP;
https://www.ptgcn.com/products/ubiquitin-Antibody-10201-2-AP.htm
Anti-NLRP3, Proteintech, Cat No. 19771-1-AP;
https://www.ptgcn.com/products/NALP3-Antibody-19771-1-AP.htm
Anti-Caspase 1, Proteintech, Cat No. 22915-1-AP
https://www.ptgcn.com/products/CASP1-Antibody-22915-1-AP.htm
Anti-COX1, HUABIO, Catalog# HA722838;
https://huabio.cn/products/MTCO1-antibody-HA722838
Anti-IgG,Proteintech,Cat No.30000-0-AP;
https://www.ptgcn.com/products/IgG-control-Antibody-30000-0-AP.htm
Anti-NEK7,BEIJING BIOSYNTHESIS,bs-7758R;
https://www.biosschina.com/#/productDetail?goods_id=8974
Anti-Flag,Sigma,F1804;
https://www.sigmaaldrich.cn/CN/zh/product/sigma/f1804
Anti-EGFP,BEIJING BIOSYNTHESIS,bs-2194R;
https://www.biosschina.com/#/productDetail?goods_id=18216
Anti-TOM20,ABclonal,A16896;
https://abclonal.com.cn/catalog/A16896
Anti-CD45,BEIJING BIOSYNTHESIS,bs-4819R;
https://www.biosschina.com/#/productDetail?goods_id=15146
Anti-4-HNE (4-Hydroxynonenal),R&D,Cat No.MAB3249;
https://www.rndsystems.com/cn/products/4-hydroxynonenal-antibody-198960_mab3249?keywords=MAB3249
Alexa Fluor 488-labeled goat anti-rabbit IgG,Beyotime,A0423;
https://m.beyotime.com/mobilegoods.do?method=code&code=A0423
Alexa Fluor 647-labeled goat anti-mouse IgG,,Beyotime,A0473;
https://m.beyotime.com/mobilegoods.do?method=code&code=A0473

Eukaryotic cell lines

Policy information about cell lines and Sex and Gender in Research

Cell line source(s)	HepG2 cells (SUNNCELL, SNL-083) and AML-12 cells (SUNNCELL, SNL-242) were originally from American Type Culture Collection (ATCC).<br>HepaRG cells were purchased from Shanghai Hong Shun Biological Technology (C0986, originally from Millipore) .<br>LX-2 cells were purchased from Procell (CL-0560).<br>The primary hepatocytes used in this study were isolated from hepatocyte NEK7 knockdown mice (the Het mice, both sex) and the WT mice (both sex).
Authentication	The cells were authenticated by short tandem repeat (STR) analysis.
Mycoplasma contamination	Cell lines were not tested, as no indication of contamination was observed.
Commonly misidentified lines (See ICLAC register)	No commonly misidentified cell lines were used in this study.

Animals and other research organisms

Policy information about studies involving animals; ARRIVE guidelines recommended for reporting animal research, and Sex and Gender in Research

Laboratory animals	Mice aged 6-10 weeks were used. The NEK7fl/fl and Alb-cre mice on C57BL/6 background were purchased from GemPharmatech (Nanjing, China) and Shanghai Model Organisms Center, respectively. The wild-type mice including C57BL/6J or BALB/c were purchased from GemPharmatech. Mice were housed in pathogen free and ventilated cages, and allowed free access to food and water in a 12h light/dark cycle, with room temperature at 22-25°C and 40-60% humidity.
Wild animals	No wild animals were used in this study.
Reporting on sex	Both sex of mice were used in the experiment related to knock-out mice. The information on sex of each individual was provided in

	Source Data and Supplementary Information. In the liver fibrosis model constructed using wild-type mice that require tail vein injection, we only selected male mice to avoid fibrosis progression that may be affected by technical deviations and the physiological estrous cycle of female mice.
Field-collected samples	This study did not involve field-collected samples.
Ethics oversight	All animal procedures were approved by the Institutional Animal Care and Use Committee (IACUC-2209063) of Nanjing Medical University.

Note that full information on the approval of the study protocol must also be provided in the manuscript.

Plants

Seed stocks	n/a
Novel plant genotypes	n/a
Authentication	n/a

Flow Cytometry

Plots

Confirm that:

- [x] The axis labels state the marker and fluorochrome used (e.g. CD4-FITC).
- [x] The axis scales are clearly visible. Include numbers along axes only for bottom left plot of group (a 'group' is an analysis of identical markers).
- [x] All plots are contour plots with outliers or pseudocolor plots.
- [x] A numerical value for number of cells or percentage (with statistics) is provided.

Methodology

Sample preparation	The cells were washed with PBS and were trypsinized for a single cell suspension.
Instrument	CytExpert
Software	CytExpert software (2.3.1.22)
Cell population abundance	For each sample, 10000 cells were recorded. The main cell population is included for analysis, with exclusion of the dead cells and doublets.
Gating strategy	The FSC-A/SSC-A gating was used to determine the main live cell population. FSC-A/FSC-H gating was used to exclude the doublets. Finally, the corresponding fluorescent signals (PE or FITC-Count) were collected for statistical analysis. Gating strategies are provided in Supplementary Fig. 12.

- [x] Tick this box to confirm that a figure exemplifying the gating strategy is provided in the Supplementary Information.
